# Optimising outcomes for complex trauma survivors: assessing the motivators, barriers and enablers for implementing trauma informed practice within a multidisciplinary health setting

**DOI:** 10.1186/s12913-022-07812-x

**Published:** 2022-04-02

**Authors:** Renee C. Lovell, David Greenfield, George Johnson, Kathy Eljiz, Sue Amanatidis

**Affiliations:** 1 Community Health Services, Sydney Local Health District, Sydney, Australia; 2 Sydney Institute for Women, Children and their Families, Sydney Local Health District, Sydney, Australia; 3 South Western Sydney Clinical School, The Simpson Centre for Health Services Research, UNSW Medicine, Sydney, Australia; 4 Australian Institute of Health Service Management, Tasmanian School of Business and Economics, University of Tasmania, Sydney, Australia

**Keywords:** Trauma informed, Healthcare, Complex trauma, Wellbeing, Multidisciplinary, Community health

## Abstract

**Background:**

Complex trauma is a significant public health issue with detrimental health, interpersonal and psychological impacts, which can impede client recovery and result in multiple representations. ‘Trauma Informed Practice’ (TIP) is an evidence-based model which ensures safe and effective services for clients and staff. This study examines health professional’s use of TIP, and the motivators, enablers and barriers to implementation in a multidisciplinary setting.

**Methods:**

A mixed methods study with 24 front-line clinicians and managers within a community health setting in Australia. A purpose designed, expert validated TIP checklist was completed, followed by semi-structured focus groups. Survey data was reported using descriptive statistics. Focus group data was digitally recorded, transcribed and thematically analysed.

**Results:**

Ten key factors were identified motivating, restricting or enabling TIP implementation. Seven were organisational factors including supportive and informed management, flexibility of service models, levels of service demands, resource availability, education opportunities, good client outcomes, and reporting requirements. Philosophical approach, team orientation, and vicarious trauma/stress management were three individual professional factors. Critically, alignment in two ways was necessary for successful implementation, that is: in knowledge and understanding across organisational role levels - clinician, manager and executive; and, in professional philosophy and team orientation of individual clinicians.

**Conclusion:**

Providing TIP is essential for ensuring optimum client outcomes for trauma survivors and for maintaining workforce wellbeing. Although the increasing uptake to TIP is evident within the health setting, further attention is required to address the tension between service models focused on efficiently servicing whole populations and those attuned to effectively meeting the needs of high risk groups. A complex strategy to unite therapeutic and managerial goals is necessary if client, professional and organisational needs are to be effectively met.

## Background

### Complex trauma

Complex trauma is a worldwide public health challenge, which leads to detrimental health, interpersonal and psychological impacts [1, 2]. Complex trauma is often experienced as interpersonal violence, sexual assault or child abuse [3], the rates of which are alarmingly high, both in Australia and internationally [3–5]. The financial cost of complex trauma to the Australian health system is estimated at $22B per year [6], a cost which increases when health services fail to make the link between complex trauma and the presenting issue [7]. Complex trauma survivors present across the whole health system, with a range of physical, psychological, and interpersonal issues [7]. These include, but are not limited to, unwanted pregnancy, disability, chronic health conditions (such as cancer, liver and heart disease) [8], depression, substance use issues, and psychiatric or personality disorders [9, 10].

However, not all trauma survivors develop problematic, long-term trauma symptoms [11]. Evidence indicates socio-economic disadvantage and the cumulative effect of multiple adverse events over time, to be the key contributors to the development of mental health issues and chronic illness for complex trauma survivors [12]. Furthermore, the interrelated factors of trauma-related neurodevelopmental changes, life stressors, and systemic discrimination, can lead to behaviours which negatively impact health, and impede healthcare engagement [11].

Many complex trauma survivors experience multiple barriers to accessing healthcare [11]. Services which fail to take into consideration complex trauma and its impacts, inadequately address underlying health issues and can be re-traumatising and disempowering to clients [11]. Clients who have experienced re-traumatisation by a health service have been found to develop a disconnection with their health worker, and a mistrust of health services [9]. As a result, they pull away from engagement and ongoing care, and access anonymised transactional care such as in emergency departments [9]. This exacerbates health issues, resulting in multiple re-presentations over a lifetime, and poorer health outcomes overall [11].

Given the high prevalence of complex trauma and the detrimental impacts of unrecognised or poorly addressed trauma [13], it is prudent that services treat all clients with sensitivity to complex trauma. This can be achieved through implementing Trauma Informed Practice (TIP), also known as Trauma Informed Care and Trauma Informed Service Delivery [13]. TIP is a safe, effective, cost efficient and evidence based model which is implemented universally into policy and practice across the health system [2, 8, 13]. TIP underlying principles are safety, trustworthiness, collaboration, empowerment, choice [8, 14], and acknowledgement of cultural, historical and gender issues [15]. The acknowledgment of cultural and historical issues is a more recent, but integral component of TIP. It means that healthcare services work to move beyond discrimination and bias [16], provide culturally informed and responsive services [16], acknowledge the importance of cultural connectedness, and that they recognise the impacts of colonisation and address historical trauma [16].

For health professionals, working with trauma survivors can negatively impact their health and wellbeing [17] and for some, be confronting as they themselves have their own experiences of trauma [17]. A forward thinking organisation utilising TIP puts systems in place to mediate against this, and to identify and respond to it when it occurs [18]. TIP offers a consistent, appropriate approach that achieves better health outcomes and satisfaction for complex trauma survivors [19], increases staff confidence and satisfaction [19], and creates better relationships between service providers and clients [19].

TIP is relevant to many services and professional groups in different healthcare settings [8, 11]. Health services that have been shown to be receptive to the introduction of TIP are inter-professional and team orientated in philosophy and norms, for example mental health [20, 21], drug and alcohol [12, 22], and primary care [11]. The use of TIP has been undertaken by health professionals from a variety of disciplines, including social work [22, 23], nursing [21, 24], medical staff in midwifery, obstetrics, and gynecology [11] and emergency medicine [25].

A clear message from previous research is that the successful implementation of TIP requires addressing systems barriers and building workforce motivation, knowledge and skills [26], with consideration of the context in which it is being implemented [19]. Despite the growing body of evidence supporting TIP implementation in Australia and internationally [2, 7, 20], the majority of TIP implementation studies within the healthcare sector have been discipline and/or departmentally specific, with a strong focus on mental health and drug and alcohol services. Evidence however is limited, within a multi-disciplinary, multi-service type, community health setting, which is outside of a mental health or drug and alcohol service context.

Hence, the study aim was to examine motivators, barriers and enablers to the implementation of TIP among multidisciplinary healthcare professionals, in a multi-service type, community healthcare setting in Australia.

## Methods

### Setting and recruitment

The study was set within four specialist community health services, based within a large hospital network in Sydney, Australia. Sydney Local Health District (SLHD) is a densely populated and socio-economically and culturally diverse region with a population of 640,000 people (2016). Community health services provide around 200,000 non-admitted patient occasions of service annually, of which around 26,000 are provided by specialist services [27]. Specialist services are out-patient services, designed to meet the non-acute health needs of those experiencing the greatest social and economic disadvantage, the highest prevalence of disease and/or the poorest health [27]. Specialist services provide medical services, case management and psychosocial services for priority populations, including young people, people who have experienced sexual assault, people with diverse sexualities and gender identities, and people living with HIV. They also provide community education, workforce capacity building and policy advice on matters relating to violence, abuse and neglect. As the majority of Specialist Services work with clients rather than patients, this is the term that has been chosen for reporting on this study. The term client aligns with the origins of TIP within the Australian health system [18, 28], whereby seeing the service user as a client, places them in a position which is considered more equal to the clinician, and where decision making is shared [29].

Services selected for participation in the study were those which met the criteria of being a clinical service and having a client base where at least 25% were considered marginalised or experiencing disadvantage. The key indicators for this being low socio-economic status, homelessness, living with a chronic illness, and/or experiencing discrimination or cultural bias. Selected services included HIV counselling and case management, sexual health clinical services, sexual assault counselling, and primary health care for young people. A researcher attended team meetings to explain the study and follow-up emails (limited to three reminders) were distributed outlining study details and calling for participants. There were 24 people who elected to participate in the study from across nursing, allied health, health promotion, front-line management and medical positions (Table 1). The participants represented a majority of the targeted workforce (69% *n* = 24/35). Of those who did not participate, there were a small group of health promotion officers who declined, stating that they did not feel the study was relevant to them as non-clinical staff. There was also a large group of nursing and a small group of medical and social work staff from the one service, who were restricted from participating by their manager, as they determined the staff could not be released from seeing clients. To ensure the service was still well represented, three key participants were recruited by email (representing nursing, social work and medical/other). Those joining the study were asked to provide written and verbal consent. The study protocol was approved by Sydney Local Health District – RPAH Human Research Ethics Committee (X16–0240).

**Table 1:** Professional grouping of participants

Professional Group	Participants n (%)
Social Work (SW)	9 (38)
Nursing	4 (17)
Service Managers	4 (17)
Allied Health (exc. SW)	3 (13)
Health Education Officers	2 (8)
Other	2 (8)
Total	**24 (100)**

### Data collection

#### Questionnaires

As participation did not require prior knowledge or use of TIP, it was necessary to develop a means for providing standardised information about the principles of TIP, as well as to determine the extent of its current use. For this purpose, a 15-min, self-administered, structured questionnaire was developed based on TIP best practice documents [18, 28, 30]. The questionnaire briefly explained and measured participant use of TIP principles of safety, trustworthiness, collaboration, empowerment, choice and gender sensitivity; in addition to workforce participation in trauma related training and support [28]. The questionnaire required participants to check a response box labelled ‘most of the time’, ‘sometimes’, ‘never’ or ‘don’t know corresponding with a list of questions, each representing one or more trauma informed practice principle. Responses of ‘most of the time’ and ‘sometimes’ indicated use within practice, whereas ‘never’ and ‘don’t know’ indicated no practice was in place. The questionnaire content was validated by two TIP experts, who confirmed its relevance, clarity, simplicity and low ambiguity.

All study participants (*n* = 24) answered the questionnaire within a focus group or mini-focus group setting. During completion, individuals had opportunities to ask questions and clarify their understanding of the process. These answers were shared with the whole group and noted by the facilitator for future use. Following completion, participants were given the opportunity to exit the study, or to remain in the focus group or mini-focus for the follow-on discussion. Questionnaire responses were collected at the conclusion. Responses were tallied and grouped into TIP principles for determining frequency of use.

#### Focus groups

Focus group discussions were considered the most appropriate method for identifying motivators, barriers, and enablers, as they allow for a robust discussion between participants and the generation of new ideas and/or perspectives [30]. Mini-focus groups, as described by Kamberelis & Dimitriadis (2005) [31], were also considered appropriate. This increased flexibility in attendance time and location, and supported the separation between front-line staff and managers.

Seven discussion groups were convened by the Principal Investigator (RL), who has training and extensive experience in running focus groups with professionals and service users. A focus group was conducted for front-line staff in each service approved for full participation. These were scheduled during a time usually allocated to a team meeting or professional education session. Attendance was optional and was not reported back to service managers. Mini-focus groups were scheduled at a time most suitable to the participants. Both focus and mini-focus groups were undertaken, using a structured guide containing questions relating to the motivators, barriers and enablers experienced by the participating workforce, within their workplace, when using, or attempting to use any of the TIP practices featured in the questionnaire. Focus groups and mini-focus groups ran for 45 min, commencing immediately following questionnaire completion. All staff members who elected to participate in the questionnaire, elected to remain for the subsequent focus group or mini-focus group discussion (*n* = 24). Of the seven groups, four were mini-focus groups of two participants (managers or key informants), and the remaining three were focus group discussions, with either five or six front line staff participating. All discussion groups were multidisciplinary, excluding one focus group which was social work specific. During the discussion, individuals were encouraged to reflect on their questionnaire responses to inform their answers. In line with best-practice as outlined by Ochieng, Wilson, Derrick, & Mukherjee (2018) [31], the convenor took on a passive role within the discussion, maintaining a neutral position, and encouraging discussion between participants, whilst also seeking clarification, controlling group dynamics, preventing dominant members from shaping the discussion, and ensuring each participant was provided an opportunity to discuss their opinions. Focus groups and mini-focus groups were recorded and transcribed by an independent service.

### Data analysis

Questionnaire responses were de-identified and collated, summarised to the service level and reported using simple descriptive statistics. Due to the small sample size, and the study focus on qualitative analysis, inferential statistical analysis was not undertaken. Focus group and mini-focus group responses were de-identified and analysed by the Principle Investigator (RL), using the constant comparative method to generate themes and meaning. As described in Bradley, Curry & Devers (2017) [32], initial codes were identified by reading and re-reading each transcript and identifying key words and emergent categories. These were then grouped according to likeness and developed into potential themes and sub-themes. Themes were then cross checked for relevance and to eliminate duplication. They were counted for total frequency and frequency across groups, to determine level of relevance across the workforce. Microsoft Excel was used as a data management tool. A senior academic (KE) provided advice and consultation, and assessed the theme development process, and subsequent findings for truth, value, neutrality, accuracy, and relevance. Findings were also reviewed for accuracy and applicability by a senior health service manager (SA). Member-checking was undertaken by sending preliminary results via email to three key informants from social work, nursing and allied health/management for review. Preliminary results were also presented to the members of the participating workforce and feedback was elicited. This was undertaken within the context of a broader research event. All feedback was used for validation and further refinement.

## Results

### Education for and reported use of TIP

Education for and use of TIP in clinical practice was reported by participants as variable (Tables 2 and 3). Participants reported training and vicarious trauma prevention education was limited. Around half of the participants indicated they had never attended a course on trauma issues (*n* = 11, 46%), and one third stated they had never received supervision where vicarious trauma was discussed (*n* = 7, 29%).

**Table 2:** Education for using TIP in clinical practice

TIP principle represented by question	Questions In this role:	Response	
		YES	NO
Safety	Have you attended training or education about the impacts of trauma?	13	11
Safety, Collaboration	Have you attended training or education about developing safety and crisis plans?	11	13
Empowerment	Do you know where to refer clients for trauma-specific services and interventions?	21	3
Empowerment, Choice	Have you attended any education or training on mindfulness?	13	11

**Table 3:** Use of TIP in clinical practice

TIP principle represented by question	Questions In your work:	Response			
		Never	Sometimes	Most of the time	Don’t know
Safety	Do you provide a physically safe environment for clients?		3	21	
Safety	Do you provide an emotionally safe environment for clients?		6	17	
Collaboration, Empowerment, Choice	When discussing service options with clients do you share decision making with them?		2	22	
Empowerment, Choice	Do you believe the way you interact with clients maximises their choice and control?		6	18	
Safety	Do you attend ongoing supervision where preventing vicarious trauma is actively discussed (within your service or externally)?	7	10	6	1
Trust, Safety	Do you receive ongoing support (separate to supervision) where preventing vicarious trauma is actively discussed?	12	10	2	
Trust, Safety	Do you provide a culturally safe service?		9	15	
Trust, Gender Sensitivity	Do you provide a gender-sensitive service?	1	2	20	
Collaboration, Safety	Do you communicate in an open and respectful manner with clients?		1	23	
Collaboration	Do you support the clients’ goals and interests?		2	22	
Empowerment	Do you refer clients to trauma-specific services and interventions if required?	3	7	12	2
Safety, Empowerment	Do you practice mindfulness in your daily work?	5	15	4	
Collaboration, Empowerment	Do you promote mindfulness as a strategy for clients?	2	16	5	1

All, bar one, participant (*n* = 23, 96%) reported using elements of TIP in client work, either sometimes or most of the time. Practices most commonly reported were: use of open and respectful communication (*n* = 23, 96%); sharing decision-making (*n* = 22, 92%); providing a gender sensitive service (*n* = 20, 92%); and supporting client goals and interests (*n* = 22, 92%).

### Motivators, barriers and enablers to TIP implementation

Participants were mostly consistent in their views across groups over the three areas explored, these being motivators, enablers and barriers to TIP implementation (Table 4). Participants explained that they were motivated to use TIP as it aligned with professional norms and enabled the achievement of good client outcomes. Four enablers to TIP implementation were highlighted by participants to do with flexibility within service models, supportive management, supportive front-line colleagues, and multidisciplinary teamwork. Similarly, participants indicated four barriers to TIP in clinical settings, including workload pressures, time demands, work management processes, and education needs. These issues are expanded upon below.

**Table 4:** Motivators, Barriers and Enablers to TIP implementation

Category	Theme	Factor Context	Responses	
			Number mentions (total)	Number groups mentioned in (*n* = 7)
Motivators	Results in good client outcomes	Personal	16	7
	Is aligned with teachings of professional group	Personal	9	5
Barriers	High levels of stress due to perceived workload pressure to meet strict performance targets	Personal	25	6
	Challenges working with clients with complex trauma and addressing their needs within allocated timeframes	Organisational	29	5
	Tensions between: activity based funding, slow recruitment and waitlists; and staff wellbeing, self-care and effectiveness	Organisational	18	6
	Difficulty accessing funding to attend training and other professional development activities	Organisational	18	7
Enablers	Flexibility and compatibility within the service model to address client needs, ie., with timeframes, ability to meet client in the community	Organisational	24	6
	Supportive and informed management of TIP at the frontline and executive level	Organisational	16	7
	Formal and informal support mechanisms with frontline colleagues, ie., debrief with fellow staff and case review meetings	Organisational	17	6
	Multidisciplinary teams allowing for efficient referrals and a team approach to addressing complex client needs	Organisational	12	5

#### Motivators

Participants expressed positive attitudes towards use of TIP in their work. The most common reason discussed was that it was best practice and resulted in good client outcomes, a view consistent from participants across services and disciplines.

*“Our clients often require ongoing therapy, with a trauma informed therapeutic relationship to work through and process the trauma ... I would say there is still that understanding that this is best practice with clients … and that to develop that relationship, you need certain things in place.”* (Social Worker, FG1)

*“And I also think from a cultural point of view it’s a part of our culture, to serve people with best practice, and to make sure that we give them choices and help support them.”* (Other FG2)

A further point reinforcing professional motivation for TIP was the fact that participants recognised that TIP, and use in practice, aligned with the norms, values and expectations of their respective professions.

*“In terms of my work, it’s all about changing behaviour … and for someone to be comfortable … you need to make sure clients feel safe, physically and emotionally … Making change is always difficult, so if they don’t feel safe our interventions not really going to happen.”* (Allied Health Worker, FG3)

*“I think for me, as a social worker, it actually embodies all the social worker values and ethics … It’s very much client oriented, and about respect for the client around their self-determination. We are working collaboratively with clients.”* (Social Worker, FG1)

#### Barriers

When asked about what made it difficult to use TIP in their work, both front-line professionals and managers, across disciplines, spoke about perceived pressure to do more due to workload measures or performance targets. Front-line professionals explained feeling ‘stressed’ or ‘under the pump’ due to service demands and reporting requirements. Participants linked their experience to the requirement to report client work against quantitative benchmarks, with limited capacity to record trust, safety, or empowerment outcomes for the client. Organisational and service management systems were not structured or equipped to deal with the challenges of delivering services to clients who have experienced complex trauma.

*“I think activity-based funding is a barrier … you might see one person and spend a day with them … but still management could come and say you’ve only seen one person … But that one day could make such an impact on their overall situation.”* (Nurse, FG2)

*“But there’s definitely something happening and I think it’s going to get worse because of our targets and our, you know, the pressure … and the more stressed we are, the more we are under the pump. You are not going to be alert to another person’s needs.”* (Other, FG4)

Similarly, front-line managers acknowledged that performance measures are an organisational reality, but also recognised the importance of looking after professional wellbeing and delivering a service that was effective for clients. Participants explained that balancing the two requirements was a continual challenge.

*“The number of client targets puts pressure on staff to do more direct client work which is what we’re there for, but it makes it harder to release them for activities that are more nurturing like health promotion or research because they’re just face to face and that’s the only focus.”* (Manager, FG7)

*“So suddenly it’s about crunching numbers, getting numbers through to get funding. So for me that’s a barrier … because they [staff] are constantly worried about workload measures … but the problem is, the tension with that model and being effective.”* (Manager, FG6)

Participants reflected upon the negative cycle that services could become trapped in, undermining the outcomes for clients and staff motivation. They reported the situation as follows: not meeting service targets could lead to a need to save money through positions being left vacant or funding for training being restricted; which in turn increased client waiting lists/times; leading to additional workload pressures for staff to see more clients; and, reduced the opportunity for staff to undertake training, self-care activities or research projects. This cycle, once established, could prove difficult to break.

*“Another one, which follows from positions not being filled, it means long waiting lists, which means you would like to follow up a [client] … you know that they’d want to come back but they need some nudging. You don’t have the time but if you’re dealing with someone who’s traumatised they need that TLC.”* (Nurse, FG2)

*“When I look back to when positions were unfilled I wonder how I didn’t actually walk out because it was actually so stressful … I think because we are not doctors, we are not seen as a priority and that’s a risk for the team.”* (Social Worker, FG2)

#### Enablers

When asked what supported their use of TIP in clinical practice, participants from all services and disciplines, discussed the importance of flexibility and compatibility within service models. Many professionals provided examples explaining that service models that integrated TIP enabled a focus on client needs, timeframes and meeting the client where they best felt comfortable. The point was expressed in these ways:

*“We can literally meet clients where they are, in a practical sense and an emotional sense … flexibility, that’s the one thing that stands out, what has enabled us to engage with people.”* (Social Worker, FG3)

*“I suppose different models of care to improve access for people … we do give people a lot of choice within the parameters. They can opt in and out of a whole variety of things here, so not everyone has to be funnelled into the same kind of service.”* (Manger, FG5)

Additionally, participants reported that having supportive managers, both at the front-line and executive level was critical. A shared understanding of the value of TIP and willingness to implement TIP in the service ensured alignment of purpose.

*“We’ve been lucky because our management has fought to push the vicarious trauma aspect of our work.”* (Social Worker, FG1)

*“It was having upper management support. For example, thinking about clinical supervision, that’s very important for us.”* (Manager, FG7)

Reinforcing the importance of support for TIP from the managerial levels, participants noted the availability of informal and formal support mechanisms from colleagues as also fundamental. They discussed the psychological and emotional support they needed and received, and offered to colleagues in return, as an important variable in effective TIP implementation. Participants explained their experiences as follows:

*“You feel supported in the team, which I think helps in your individual work and your work with the clients. Sometimes you need to debrief … and if you didn’t … it would have an impact on ability to work with clients.”* (Social Worker, FG3)

*“I find case review meetings great, because not coming from the clinical background, I’m able to see how the counsellor or the nurse worked with a client and it helps me get ideas for if I face that.”* (Other, FG2)

A further enabler to TIP implementation was working in multidisciplinary teams. Participants stated they enabled immediate collaboration, support, enhanced referral processes, and the additional benefit of cross disciplinary learning.

*“I think it is the culture here. So it creates a safe nursing space because you can keep on time with the rest of the clinic work, and get patients into a service you know is good quality without having to do it yourself. It’s pretty unique to be able to book someone into a counsellor. It makes it much easier … to feel more comfortable about asking a bit more because referrals is so easy.”* (Nurse, FG4)

*“The different specialities within the team have allowed us to do more … like, I think health education is a really important component of the work … and there’s the work of the Aboriginal Health Education Officer with Aboriginal communities that increases our services engagement with them. The thing that allows us to do all of this is that we are a multidisciplinary team.”* (Social Worker, FG3)

## Discussion

This study is an examination of the motivators, barriers and enablers to the implementation of TIP in a multidisciplinary, multi-service type, non-acute, outpatient, community health setting. It has identified ten key factors motivating, restricting and enabling the implementation of TIP in practice. These factors may be conceptualised as related to the organisational or service context, or individual professional. Seven factors are related to the organisational or service context, including: supportive and informed management; flexibility of service models; levels of service demands; resource availability; education opportunities; good client outcomes; and reporting requirements. Three factors are associated with the individual professional delivering the service, including: philosophical approach; team orientation; and vicarious trauma/stress management. Critically, alignment in two ways was deemed necessary for successful TIP implementation, that is: in knowledge and understanding across organisational role levels from clinicians, to managers, to executives; and, in professional philosophy and team orientation of individual clinicians.

This study confirms previous research in other health settings, including drug and alcohol, mental health, emergency medicine, and paediatric and trauma nursing [11, 24, 25, 33]. Additionally, it supports findings from discipline specific studies in nursing, social work, medicine and midwifery [2, 24, 25]. In particular, as seen in studies in emergency medicine and paediatric nursing, participants across disciplines consistently spoke about their services’ existing capacity for TIP, in multiple service entry points, ensuring clients had choices, collaborative goal setting and treatment processes, with seamless referral pathways, and open and transparent communication [24, 25]. They also noted successful implementation of trauma informed systems, such as supporting peer debriefing, case discussion, clinical review and cross disciplinary learning. This was consistent with study findings and best practice documents in the mental health setting [18, 21, 28, 34].

As seen in studies examining the implementation of TIP within emergency medicine, mental health, paediatric and trauma nursing, a number of barriers were noted. In particular, lack of trauma informed practice at a leadership level, limited opportunities for wide-spread staff training, and the need to better inform decision makers about complex trauma [11, 24, 25]. Recommendations were made to increase opportunities to manage vicarious trauma across disciplines and to increase opportunities for trauma specific training [11, 24, 25]. It is important however to note that the limited access to trauma-specific training and supervision reported by the participants in this study, may be a result of trauma-specific training not being prioritised for this workforce due to a lack of awareness by planners/managers regarding its applicability across disciplines and service types. This has been highlighted by Hoysted and colleagues (2017) [25] as a common barrier across the health system, noting that trauma related training and supervision is traditionally the territory of social work and for this reason may be overlooked in service planning for other professional groups [25].

The study’s significant contribution to the knowledge base is demonstrating that effective implementation of TIP is achieved through attending to the inseparability between the motivators, barriers and enablers. To phrase this in the terms used above, the study highlights the ways in which the organisational or service context promotes, or restricts, the use of TIP for individuals and teams, both individually and collectively. Furthermore, it emphases the impact the orientation of the individual professional or team involved has in supporting or being hesitant to TIP implementation. In a positive or negative reinforcing cycle, organisational context influences individual and team motivation for TIP, and, in return, individuals and teams shape the context by advocating for or against TIP.

Despite the demonstrated compatibility of TIP with the majority of identified organisational and individual professional contexts within the community health setting, barriers related to funding pressure and the bio-medical model were commonly identified. Whilst this has not been explicitly highlighted in previous research on implementing TIP, it does align with the drive to use minimal resources, which was identified as a barrier in a number of TIP studies, particularly within the mental health and emergency medicine settings [11, 25]. It also aligns with findings outside of the TIP research field, whereby the ways in which health systems are financed is frequently reported as a barrier across the healthcare setting [2, 7]. This is particulary evident in regrds to providing accessible and appropriate prevention, treatment and recovery services for those who experience disadvantage, including complex trauma [35, 36].

Public health services are traditionally based on a bio-medical model delivered within a complex, large scale organisation [37]. In this context executives, managers and front-line professionals often consider service delivery differently, resulting in an inbuilt tension and diverging focus between roles. Typically, those in managerial and planning roles focus on whole populations who seek access to services, while front-line clinicians’ attention is directed to the needs of the individual.

As seen within the multi-service, non-acute, outpatient community health context, the complexity of implementing TIP across the health system is shaped by a range of factors, including: organisational inertia to change; the rigidity of the traditional biomedical model; caution with the use of resources; adherence to strict privacy requirements; limited opportunity for wide-spread staff training; and inadequate or inconsistent knowledge and understanding of TIP across professionals at all levels [2, 11, 37]. Implementing system-wide strategies to overcome these challenges is essential as service delivery which fails to take into account complex trauma impacts, risks continuing to provide fragmented, ineffective and harmful services [38]. System-wide changes to further facilitate the necessary organisational and professional cultural shift towards TIP, include changes to the way health services are funded, organised and measured [18, 19], and in the way health professionals (across disciplines) are trained, developed and supported [7, 19, 39].

Balancing health service funding criteria and performance targets, so to deliver a service accessible for a population, and the needs of individuals affected by complex trauma remains an ongoing challenge [22]. Depending on the position of the professional – executive, manager, planner or clinician – a different emphasis is focused upon. This study however demonstrates that these need not be mutually exclusive, as participating managers recognised the imperative for balancing funding and performance targets with effective care for high risk populations and workforce.

Clients with experience of complex trauma can fail to attend appointments, or when they do find it difficult to engage with clinicians and may require flexible or longer appointments, and additional case management or care coordination [18, 28, 35]. Front-line clinicians and managers therefore argue for increased flexibility in service delivery and greater attention to the needs of disadvantaged or minority populations [11]. Funding and reporting requirements focused on client numbers however remains a high priority for managers and executive for ensuring service capacity; however, these requirements limit value for individual clients and increase clinician stress levels.

Hence, the critical question: how to develop organisational systems which support trauma survivors and staff wellbeing, whilst delivering timely, effective and efficient care to the entire population? It is important that executive, managers, planners and clinicians continually address this question in a collaborative, cooperative manner, so as to avoid potential tension, conflict, and mistrust that can arise between roles with competing priorities. In the absence of trauma informed changes in health service funding models; identifying, evaluating and researching how to effectively address this challenge beyond reducing TIP to aesthetic changes, one-off training sessions and brief interventions is a future task. This is of particular importance within the COVID-19 context which continues to create stress and uncertainty, for both clients, their families and clinicians [20].

### Study limitations

There were several limitations to this study. Firstly, there was a potential for selection bias, as participants self-selected to participate, and therefore it is possible that those who were supportive of moving toward the implementation of trauma informed practice were more likely to opt-in. In addition, the relatively small sample size limited the scope for statistical analysis, as well as the generalisability of the findings more broadly. However the cross-discipline approach and high level of participation from the selected sample helps to mitigate this.

Secondly, it is important to note that the data collection tool (questionnaire) for determining the level of use of TIP by the workforce does not provide comprehensive coverage of all the elements of trauma informed practice, as it was designed with the intention of providing education and capturing a snapshot only. The definitions provided on the tool, were limited as was the time allocated to tool completion. Furthermore, whilst the tool was validated for relevance, clarity, simplicity, and low ambiguity, by two TIP experts, it was not a validated instrument. This is identified as an area for future research. Additionally, potential exists for user bias towards over reporting based on what the user would prefer to be doing (or considers the right thing to do) rather than what they are actually doing (in their practice). However, the study helps to reduce the limitations of the questionnaire tool by completing in-depth explorative focus groups and mini-focus groups which were independently transcribed and analysed by the Principal Investigator (RL), who immersed themselves in the data, and completed the qualitative analysis with consultation and support from a senior academic, and a senior health manager, who assessed the findings for accuracy, truth value, neutrality, relevance and applicability.

Finally, the study was conducted within an outpatient community health setting, and therefore cannot be considered as indicative of the motivators, barriers or enablers within the inpatient, and/or hospital setting.

## Conclusion

Providing TIP is essential for ensuring optimum client outcomes for trauma survivors and for maintaining workforce wellbeing. In order to effectively implement TIP, managers and service planners must attend to the interconnected motivators, barriers and enablers that exist within a healthcare organisation. However, further attention is required to address the tension between service models focused on populations and those on high risk groups. The alignment of the majority of the organisational and individual professional contexts with TIP, within the community health setting, creates fertile ground for effectively resolving this tension. A complex strategy to unite therapeutic and managerial goals, targeted differently in specific settings, across all levels of a healthcare organisation is necessary if client, professional and organisational needs are to be effectively met.

## Data Availability

The data generated and/or analysed during the current study are available from the corresponding author on reasonable request.
